# A Low-Cost, Hands-on Module to Characterize Antimicrobial Compounds Using an Interdisciplinary, Biophysical Approach

**DOI:** 10.1371/journal.pbio.1002044

**Published:** 2015-01-20

**Authors:** Karishma S. Kaushik, Ashley Kessel, Nalin Ratnayeke, Vernita D. Gordon

**Affiliations:** 1 Department of Molecular Biosciences, University of Texas, Austin, Texas, United States of America; 2 Center for Nonlinear Dynamics and Department of Physics, University of Texas, Austin, Texas, United States of America; 3 Institute of Cellular and Molecular Biology, University of Texas, Austin, Texas, United States of America; Stanford University, UNITED STATES

## Abstract

We have developed a hands-on experimental module that combines biology experiments with a physics-based analytical model in order to characterize antimicrobial compounds. To understand antibiotic resistance, participants perform a disc diffusion assay to test the antimicrobial activity of different compounds and then apply a diffusion-based analytical model to gain insights into the behavior of the active antimicrobial component. In our experience, this module was robust, reproducible, and cost-effective, suggesting that it could be implemented in diverse settings such as undergraduate research, STEM (science, technology, engineering, and math) camps, school programs, and laboratory training workshops. By providing valuable interdisciplinary research experience in science outreach and education initiatives, this module addresses the paucity of structured training or education programs that integrate diverse scientific fields. Its low-cost requirements make it especially suitable for use in resource-limited settings.

## Abstract 2

A cost-effective and resource-efficient hands-on educational module that uses an interdisciplinary approach to characterize antimicrobial compounds, combining microbiology experiments and a physics-based analytical model.

This Community Page is part of the Education Series.

## Introduction

Antibiotic resistance is a major public health problem worldwide [1–3]. Increased resistance of bacteria to current antibiotics and a steady decline in the development and approval of newer antibiotics [4, 5] motivate efforts to search for novel antimicrobial agents, especially from natural sources [6–9]. As part of a science outreach initiative, we have developed a hands-on experimental module to study and characterize antimicrobial compounds. This module was first implemented at the Hands-On Research in Complex Systems School held at the Abdus Salam International Centre for Theoretical Physics (ICTP) in Trieste, Italy, in July 2014 [10].

The Hands-On Research in Complex Systems School aims to introduce young scientists from developing countries to cutting-edge science in physical, chemical, and biological systems [10]. These 2-week-long schools focus on using simple, low-cost, and reliable techniques to perform tabletop research in daily, 3-hour-long laboratory sessions. In these sessions, small groups of 4–6 participants work closely with two instructors, who conduct the same laboratory session each day with a different set of participants. Typically, participants either have or are pursuing advanced degrees in a physical science or engineering field. More than half of the participants are theorists or simulators, not experimentalists. Instructors usually develop a hands-on module based on a research project being pursued in their lab and tailor it to the requirements of this science outreach initiative. Specifically, the module should last 3 hours and cost $150 for 60 participants over 10 days.

The aim of the antibiotic resistance module is to introduce basic experimental microbiology concepts to an audience unfamiliar with biology and to teach students to integrate physics-based quantitative analysis with biological data. Participants test the antimicrobial activity of different compounds to gain insights into the diffusive behavior, and thereby the physical size, of the active ingredient. The laboratory module is designed to be conducted over a 3-hour time period, consisting of four main sections: overview of the scientific problem, introduction to experimental equipment and techniques, hands-on experiments, and application of experimental data to an analytical model (Box 1).

**Box 1. Overview of the Interdisciplinary Hands-on Module**

- Brief overview of the scientific problem
  - The problem of antibiotic resistance
  - Need to search for novel antimicrobial agents
  - Gaining insights into the active ingredient could help develop a therapeutic formulation
- Introduction to experimental techniques to be used
  - Participants watch short video clips of experimental techniques and tools used in the module.
  - The videos, created by us, included preparation of media and equipment, experimental design and execution (disc diffusion assay), and analysis of results.
- Performing hands-on experiments
  - The assistant instructor demonstrates the experimental steps to the group.
  - Following this, each participant performs the experiment individually.
  - Results from the previous days’ experiments are read.
- Application of the experimental data to an analytical model
  - An analytical model is used to probe the physical characteristics of the active antimicrobial component.
  - Participants watch video clips of additional experiments related to the analytical model.
  - Data from biological experiments are used to determine the diffusion coefficient and molecular weight of the active ingredient.

### Overview of the Scientific Problem

Before the sessions, all participants received a handout describing the experimental module (S1 Text). In the first section, the instructor provided a brief overview of the global problem and challenges of antibiotic resistance and the imperative need to discover novel antimicrobial compounds. This opened a brief discussion on recent efforts towards exploring natural, biological, and indigenous resources as antimicrobial agents [6–9]. Importantly, this overview emphasized the key feature of our laboratory module, whereby an interdisciplinary approach could aid the discovery and identification of novel antimicrobial compounds, especially those from natural sources.

### Introduction to Experimental Equipment and Techniques

In the second section, participants were introduced to the experimental equipment and techniques used in the module (S1 Text). We used the disc diffusion assay to test the antimicrobial activity of different compounds [11, 12], a technique used in clinical and research microbiology laboratories to study the bacterial and fungal strains’ susceptibility to antibiotics [13, 14]. In the assay, compounds to be tested for antimicrobial activity are deposited on filter discs that have been placed on a lawn of bacterial cells. The perimeter at which the concentration of the compound falls below the inhibitory threshold defines the edge of the “zone of inhibition” in which the lawn does not grow [15, 16]. The size of this zone is set by the efficacy of the antimicrobial compound against the bacterial strain on the lawn and the active ingredient’s diffusion.

We created short video clips (S1–S9 Videos) to help familiarize participants with the experimental tools and techniques. Before the hands-on experiment section, participants were introduced to the principles of sterilization, techniques for sterilization of media and equipment, methods of differentiating sterile from nonsterile media, proper use of personal protective wear, precautions to take while working with a flame, levels of biosafety risk groups and biological containment, standard operating procedures for disposal of biological waste, and appropriate handling of biological waste spills. We reinforced these concepts at the end of the module and emphasized the importance of close coordination with biological safety personnel.

### Hands-on Experiments

The hands-on sessions were conducted in improvised classrooms, so we used a risk group 1 (biosafety level 1) organism [17, 18]. The bacterial strain, growth media, and antimicrobial compounds used are listed in Table 1 along with the associated references [19–27]. Briefly, we used *Escherichia coli* DH5 *a*, a commercial broth, and eucalyptus oil, ethanol, and hydrogen peroxide as antimicrobial compounds.

**Table 1:** Bacterial strain, media conditions, and antimicrobial compounds.

**Bacterial strain**	*E. coli* DH5*α*	Low-risk, nonpathogenic, laboratory strain.Classified as a risk group 1 (biosafety level 1) organism [17].
**Bacterial growth media**	LB broth and agar	0.5% yeast extract, 1% tryptone, and 1% sodium chloride, with 1.2% agar for plates [19, 20].
**Antimicrobial compounds**	Eucalyptus oil, ethanol, and hydrogen peroxide	Eucalyptus oil, a natural extract, is reported to have antimicrobial activity [21–26]. Ethanol and hydrogen peroxide are widely used for their antibacterial effects [27].

**Figure 1:**
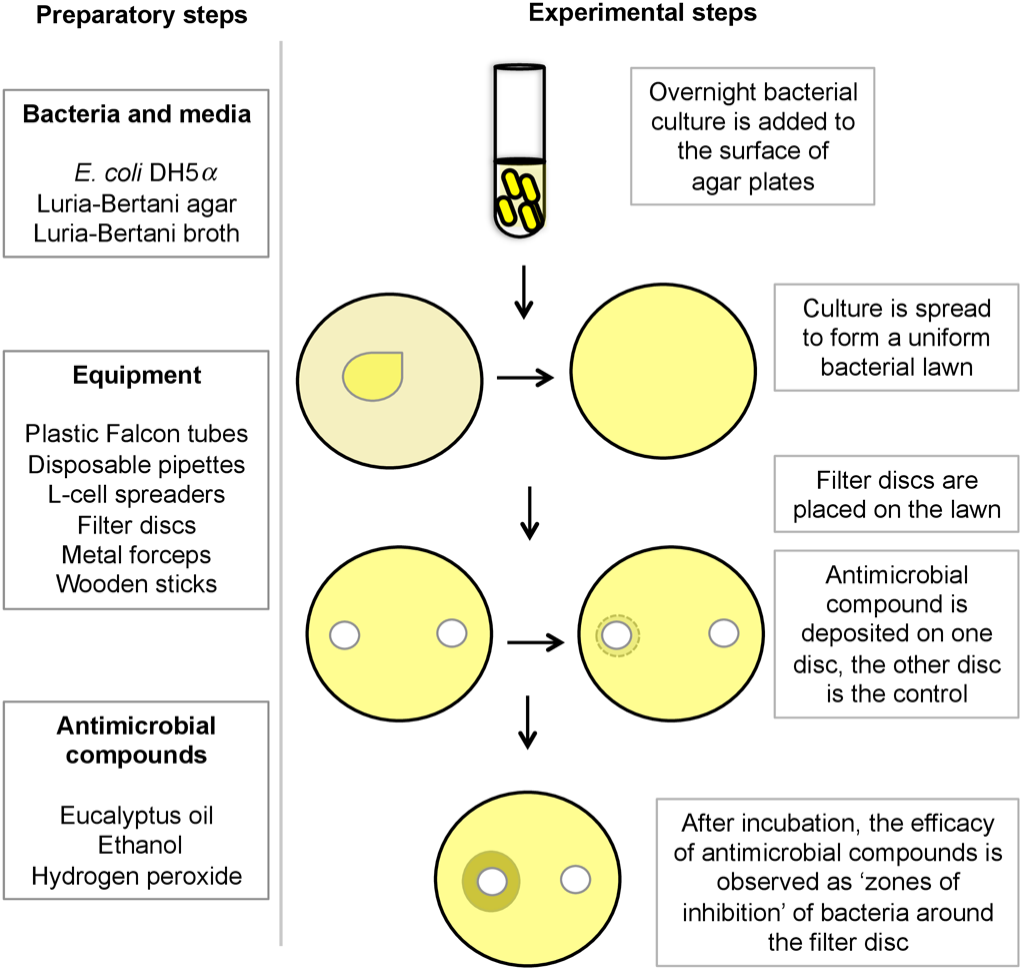
General scheme for the hands-on laboratory experiments. Preparatory and experimental steps of the disc diffusion assay to test antimicrobial efficacy.

At the start of the hands-on section, the assistant instructor demonstrated the experimental steps of the disc diffusion assay, and then each participant performed the assay. The experimental scheme for the hands-on laboratory section is described in Fig. 1. Three days before the sessions started, *E. coli* DH5*a* was streaked onto Luria-Bertani (LB) agar and allowed to grow at room temperature for 24–48 hours. A day prior to each session, overnight bacterial cultures were grown in 10 mL LB broth in plastic Falcon tubes (50 mL). Cultures were incubated under static conditions at room temperature for 16–18 hours. (The facilities available included neither a shaker nor incubator.) We inoculated overnight cultures for the first session, and participants inoculated cultures for all the other sessions. Since bacterial lawn growth and zones of inhibition would be visible only after overnight incubation, participants used experimental plates from the previous days’ session to interpret the results of the assay.

**Figure 2:**
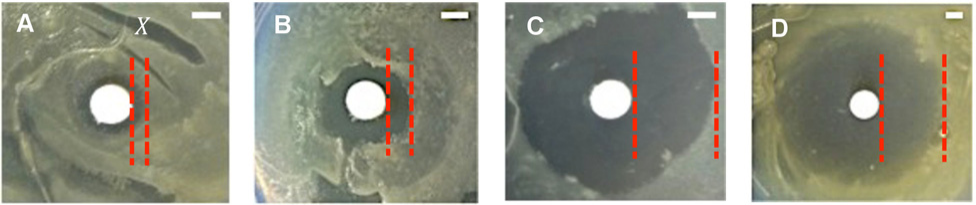
Representative zones of inhibition with different antimicrobial compounds. Using the disc diffusion assay, representative zones of inhibition observed with (A) eucalyptus oil (*X* = 2 mm), (B) 70% ethanol (*X* = 5 mm), and (C) 3% hydrogen peroxide (*X* = 14 mm) on lawns of *E. coli*. Note that these are plates prepared by participants with no previous experimental biology experience. In (D), the zone of inhibition observed with the antibiotic tobramycin (10 μL of 50 mg/mL) is shown (*X* = 18 mm). Tobramycin was used as the molecular weight standard to calibrate the molecular weight of the test compound. Owing to budgetary constraints, participants could not be provided with antibiotics for testing purposes. This image is from the assay performed by the instructors to measure slope and critical time. For the analytical model, the width of inhibition (*X*) was measured as the distance from the edge of the disc to the edge of the zone. Scale bars are 5 mm.

Participants noted the presence or absence of zones of inhibition caused by the test antimicrobial compounds and control and measured the width of the inhibition zone (*X*) as the distance from the edge of the disc to the edge of the zone (Fig. 2). Temperature of incubation, density of the bacterial lawn, bacterial strain used, and concentrations of the antimicrobial compound all influence the width of the inhibition zone. Differences in inhibition zone size allowed for comparison between the efficacies of different antimicrobial compounds and susceptibility profile of the bacterial strain. (Any open-access image analysis software (such as ImageJ) could be used to measure the inhibition zones [28].) Participants correctly determined that hydrogen peroxide was the most efficacious against the bacterial strain used, followed by ethanol and then eucalyptus oil.

At the end of each experimental session, we discussed biological safety and biohazardous waste disposal, including how these practices could be implemented at the participants’ home institutions. Following each session, bacterial cultures and agar plates were collected in designated bags for biological waste (with the biohazard symbol), treated with 10% household bleach (5.25% sodium hypochlorite) for 30–60 minutes [29], and then disposed of in the sink or trash.

### Application of Experimental Data to an Analytical Model

An analytical model was employed to determine the physical characteristics of the active antimicrobial component as a way to prospect for novel antimicrobial agents, in which insights into the active ingredient would aid the development of a therapeutic formulation. This model is based on the disc diffusion assay [15, 16] and assumes that the active ingredient has a constant diffusion coefficient *D* and that a threshold concentration of the active ingredient is required to inhibit bacterial cells. The number of cells in the lawn increases as the lawn incubates, which decreases the per-cell concentration of the active ingredient, which therefore no longer causes inhibition after a critical time *T_c_* of incubation. The model describes the width of the inhibition zone (*X*) as

$$X^{2}=4DT_{c}\ln(C_{0})+F(D,T_{c},C_{c})$$ (1),

in which *C*_0_ is the concentration of the antimicrobial compound deposited on the filter disc. *F* is a function independent of *C*_0_. *C_c_* is the lowest concentration of the active ingredient required to cause measurable inhibition. The slope of *X* ^2^ as a function of ln(*C*_0_) gives *D*, if *T_c_* is known. We relate *D* to the molecular weight (*MW*) of the active ingredient using the Stokes-Einstein equation [30], thus:

$$MW_{I}=MW_{A}{\left(\frac{D_{A}}{D_{I}}\right)}^{3}$$ (2),

in which *A* is the active ingredient and *I* is a known molecule that empirically calibrates the relationship between diffusion and molecular weight.

**Figure 3:**
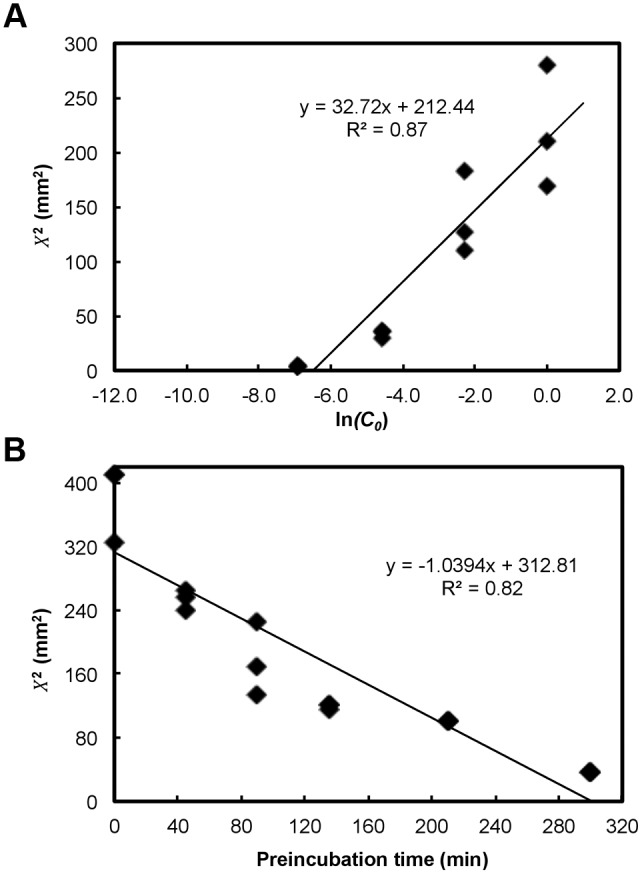
Measuring slope of *X* ^2^ as a function of ln(*C*_0_) and critical time (*T_c_*) of preincubation for hydrogen peroxide. (A) To measure the slope of *X* ^2^ as a function of ln(*C*_0_), decreasing concentrations of hydrogen peroxide are deposited and the width of inhibition (*X*) for each concentration is measured. Using linear regression, the slope of *X* ^2^ as a function of ln(*C*_0_) is obtained. (B) To measure preincubation time, a given concentration of hydrogen peroxide (30%) is deposited on the bacterial lawn after different time intervals of incubation. After overnight incubation, the width of inhibition (*X*) for each time point is measured. Using linear regression, the time after which no inhibition is observed (*X* = 0 mm) is determined as the critical time (*T_c_*) of preincubation.

To measure the slope of *X* ^2^ as a function of ln(*C*_0_), decreasing concentrations of the antimicrobial compound are deposited on bacterial lawns. After overnight incubation, the width of inhibition (*X*) for each concentration is measured. Using linear regression, the slope of *X* ^2^ as a function of ln(*C*_0_) is obtained (Fig. 3A). To measure preincubation time, a given concentration of the antimicrobial compound is deposited on the bacterial lawn after different time intervals of incubation. After overnight incubation, the width of inhibition (*X*) for each time point is measured. Using linear regression, the time after which no inhibition is observed (*X* = 0 mm) is determined as the critical time of preincubation (Fig. 3B). Since these experiments are essentially variations in the disc diffusion assay, the hands-on session in the module would provide participants the requisite skills to perform these assays. Because time was limited to 3 hours total in one morning, participants used data we had taken in our research lab. Experiments were demonstrated using video clips (S8, S9 Videos), and the participants were guided through each step in the assay. Data for hydrogen peroxide (test antimicrobial compound) and the known antibiotic tobramycin (*MW* = 467.5 Da) were provided to the participants (S1 Text and S1–S4 Tables). Using a linear regression tool with which they were already familiar (such as Microsoft Excel), participants determined the diffusion coefficient and molecular weight of the test antimicrobial compound. The handout (S1 Text and S1–S4 Figs.) also contained the solutions to the exercise.

### Participant Feedback

We did not conduct formal evaluations, but based on verbal feedback (Box 2), the participants found the module to be educational, scientifically challenging, engaging, and interesting. As physical scientists, they had almost no prior familiarity with biological experiments, and none had ever performed the disc diffusion assay. They found the choice of the scientific problem—antibiotic resistance—a relevant, relatable issue and appreciated the ease of performing the disc diffusion assay, which helped sustain their interest and boost their confidence. As theoretical and quantitative scientists, almost all of them were familiar with the laws of diffusion and diffusion-based analytical models. However, several participants reported that they had never thought of applying these conventional physical concepts to a biological problem. Some participants also said that the module provoked them into thinking of biology as a quantitative discipline in contrast to standard school and undergraduate curricula, which often teach biology as a purely descriptive science.

**Box 2. Participant Responses**

“The session was very interesting. I have heard of antibiotic resistance and worked with analytical models but never thought that I could merge the two by applying physics to study this problem.”—PhD candidate (Physics) from the Philippines

“This module was listed as my top choice as I have been offered a post-doc in a quantitative biology lab which works with yeast. This exercise has given me the confidence to work with biological organisms and assured me that my scientific training has a place in biology.”—PhD (Mathematics) from Italy

“I appreciate the effort put into mak[ing] this module exciting and informative. I always thought of biology experiments as complicated and expensive, requiring extensive skill. I never thought I would perform a biological assay, much less one that was both time- and cost-effective.”—PhD (Physics) and government scientist from Cameroon

“I am an advocate for open-source science in Mexico and can see myself using this module as a teaching tool for school children in rural and peripheral parts of my country. However, given that I do have some basic biology experience, I would have liked to have actually performed the experiments to measure slope and critical time for the analytical model.”—PhD candidate, synthetic biologist, and science advocate from Mexico

In one session, participants with no basic biology background had notable difficulty in understanding the science. This resulted in a substantial gap between their baseline knowledge and rudimentary concepts needed to understand this module. For example, one of the participants had never encountered the biological concept of a cell. While we did try to briefly address this lacuna in knowledge once we realized it, all the logical leaps that followed could not be explained in the limited time available. In subsequent implementations of this module, this could be overcome with a short, concise overview of basic biological concepts such as structure of a bacterial cell, bacterial growth and physiology, and cell death.

This was the first time this module was implemented as a science outreach tool. As a result, the logistics of executing the module in a resource-limited setting posed formidable challenges, such as shipping delays, a classroom and building not intended for biological work, and tailoring the program to suit the available budget. Owing to this, we did not focus on conducting a systematic assessment of the impact of this module on the participants. In the future, we plan to incorporate an evaluation step into this module by implementing the use of pre- and postsession surveys (using brief questionnaires) to gain a quantitative estimate of the outcome of this module on participants’ knowledge and attitudes about biophysical research. The questionnaire could be designed to not only measure participants’ understanding of the specific material presented (e.g., the problem of antibiotic resistance, potential modifications of the disc diffusion assay, and incorporating additional parameters into the analytical model) but also to gauge changes in their perceptions of larger concepts such as application of quantitative techniques to biological data and use of interdisciplinary approaches to address scientific challenges.

## Conclusions

Taken together, the features of this hands-on module make it particularly suited to science outreach in resource-limited settings such as field areas, STEM camps, high- and middle-school programs, workshops, and laboratory training exercises. This is valuable not only to developing and emerging countries but also to developed countries, where scarcity of science funding is constraining science education and outreach initiatives. Given the lack of structured training opportunities that bridge scientific fields, this module can provide valuable interdisciplinary research experience in these settings.

## Supporting Information

S1 Fig Slope of *X* ^2^ as a function of ln(*C*_0_) for compound *I*. (TIFF)

S2 Fig Preincubation time *T_c_* for compound *I*. (TIFF)

S3 Fig Slope of *X* ^2^ as a function of ln(*C*_0_) for tobramycin. (TIFF)

S4 Fig Preincubation time *T_c_* for tobramycin. (TIFF)

S1 Table Increasing concentrations of compound *I* (hydrogen peroxide) and corresponding sizes of zones of inhibition (*X*). (DOCX)

S2 Table Increasing time of preincubation and corresponding sizes of the zones of inhibition (*X*) for compound *I* (hydrogen peroxide). (DOCX)

S3 Table Increasing concentrations of tobramycin and corresponding sizes of zones of inhibition (*X*). (DOCX)

S4 Table Increasing time of preincubation (*T_c_*) and corresponding sizes of the zones of inhibition (*X*) for tobramycin. (DOCX)

S1 Text Session handout of the laboratory module. This handout was made available to participants before the hands-on session, using Google Drive. It contains more detailed information than we were able to go into during the 3-hour laboratory session, as well as links to all videos.
(DOCX)

S1 Video Preparation of broth media for bacterial growth. This video shows liquid LB media being prepared for bacterial growth. It was shown as part of the hands-on session since there were neither time nor facilities to prepare sterile media at the Hands-on School itself.
(M4V)

S2 Video Preparation of solid media for bacterial growth. This video shows agar LB media being prepared for bacterial growth. It was shown as part of the hands-on session since there were neither time nor facilities to prepare sterile media at the Hands-on School itself.
(M4V)

S3 Video Sterilization of tools. This video shows tools being sterilized. It was shown as part of the hands-on session since there were neither time nor facilities to sterilize tools at the Hands-on School itself.
(M4V)

S4 Video Preparation of filter discs. This video shows filter discs being made and packaged for subsequent sterilization. It was shown as part of the hands-on session since there were neither time nor facilities to sterilize discs at the Hands-on School itself.
(M4V)

S5 Video Preparation of bacterial strains for shipping. This video shows bacterial strains being prepared for shipping. This was not shown during the hands-on session but was available as a link through the handout.
(M4V)

S6 Video Inoculating bacterial cultures. This video shows bacteria being inoculated for growth. It was shown as part of the hands-on session. Then the assistant instructor demonstrated inoculation in person, and then each participant inoculated bacteria.
(M4V)

S7 Video Performing the disc diffusion assay. This video shows the disc diffusion assay being performed. It was shown as part of the hands-on session. Then the assistant instructor demonstrated the disc diffusion assay in person, and then each participant performed the assay.
(M4V)

S8 Video Experiment to measure the slope of *X* ^2^ (square of the width of inhibition) as a function of ln(*C*_0_). This video shows the experiment, which was based on the disc diffusion assay and measures the relationship between the width of the zone of inhibition and the amount of inhibitory material deposited. First the instructor presented the analytical model, then this video and S9 Video were shown during the hands-on session, and finally example data obtained from this experiment were made available to students for fitting to the model.
(M4V)

S9 Video Experiment to measure preincubation time *T_c_*. This video shows the experiment, which was based on the disc diffusion assay and measures the critical preincubation time. First the instructor presented the analytical model, then S8 Video and this video were shown during the hands-on session, and finally example data obtained from this experiment were made available to students for fitting to the model.
(M4V)
