# Supplementary figures and images for: A Low-Cost, Hands-on Module to Characterize Antimicrobial Compounds Using an Interdisciplinary, Biophysical Approach
Source: PLoS Biol. 2015 Jan 20;13(1):e1002044. doi: 10.1371/journal.pbio.1002044 (PMC4300086; doi:10.1371/journal.pbio.1002044)

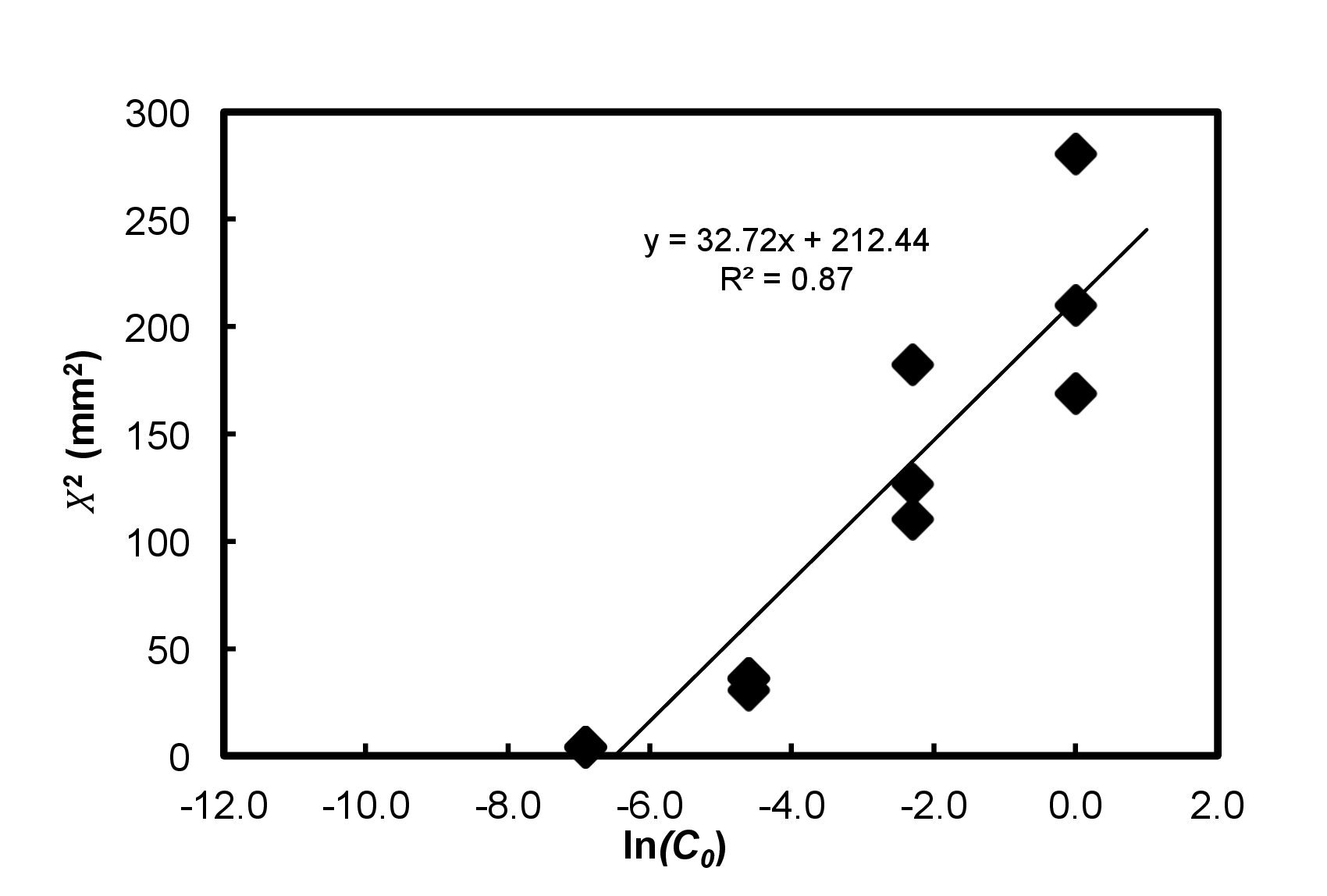

Supplement: S1 Fig — (TIFF) [file pbio.1002044.s001.tiff]

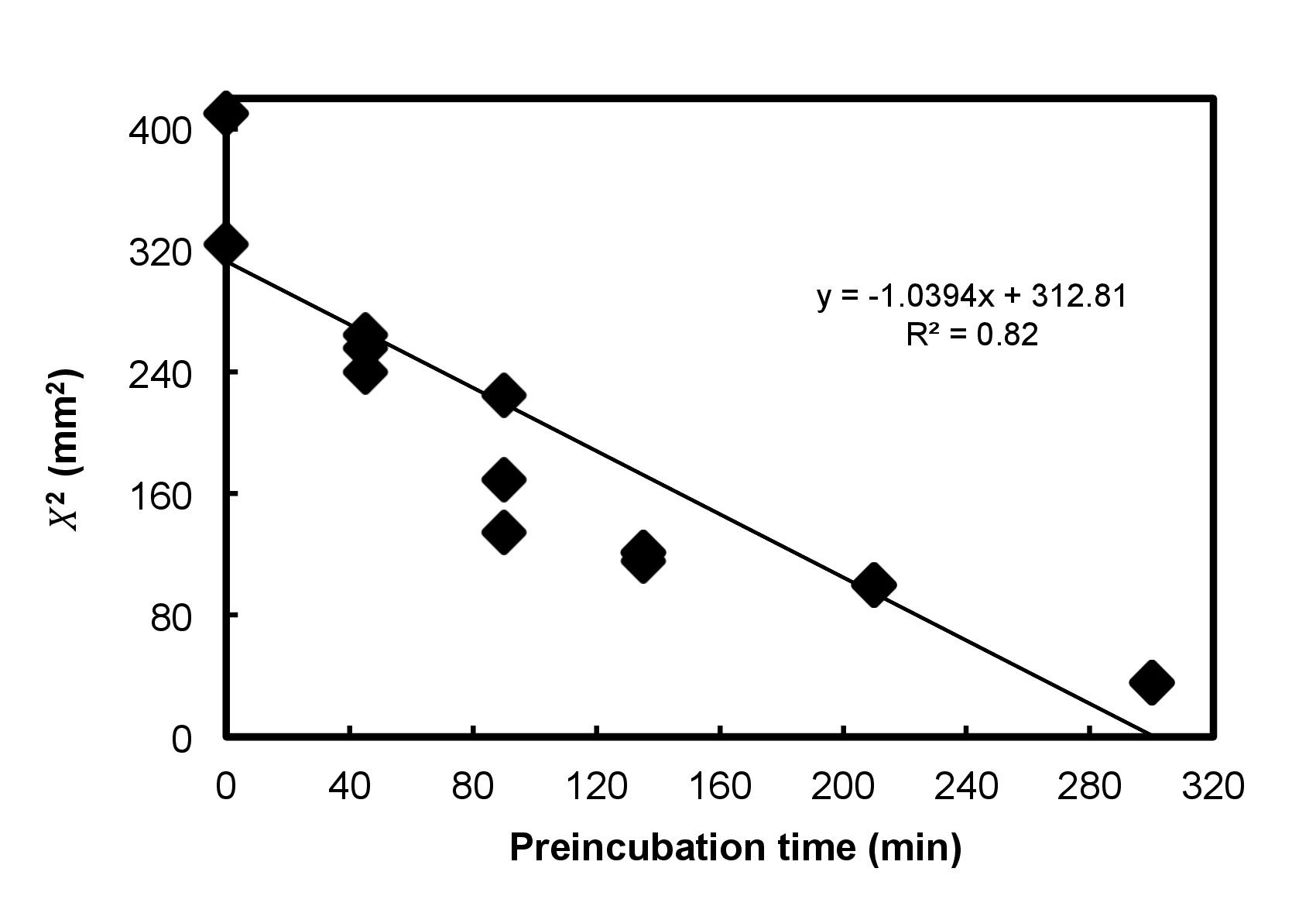

Supplement: S2 Fig — (TIFF) [file pbio.1002044.s002.tiff]

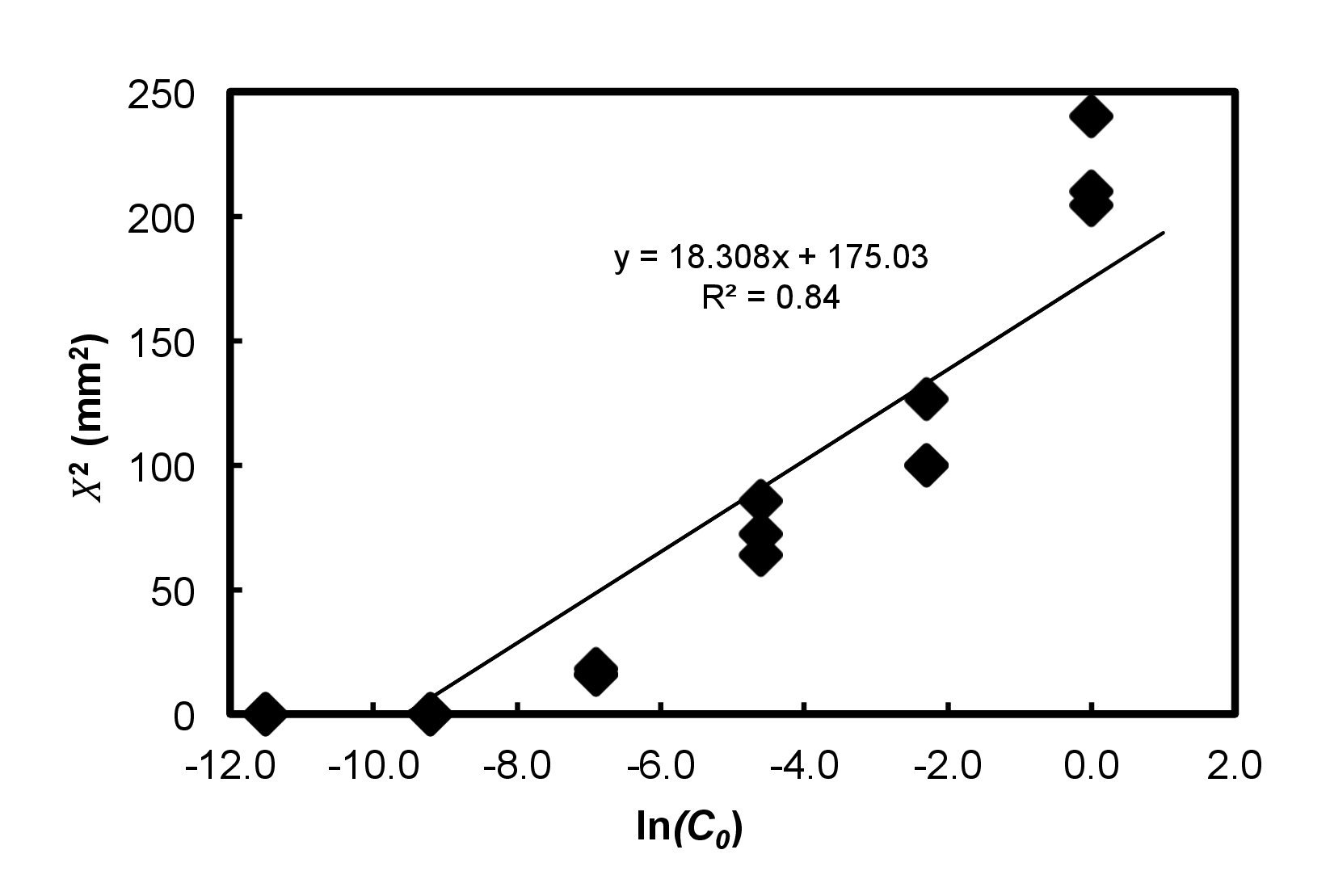

Supplement: S3 Fig — (TIFF) [file pbio.1002044.s003.tiff]

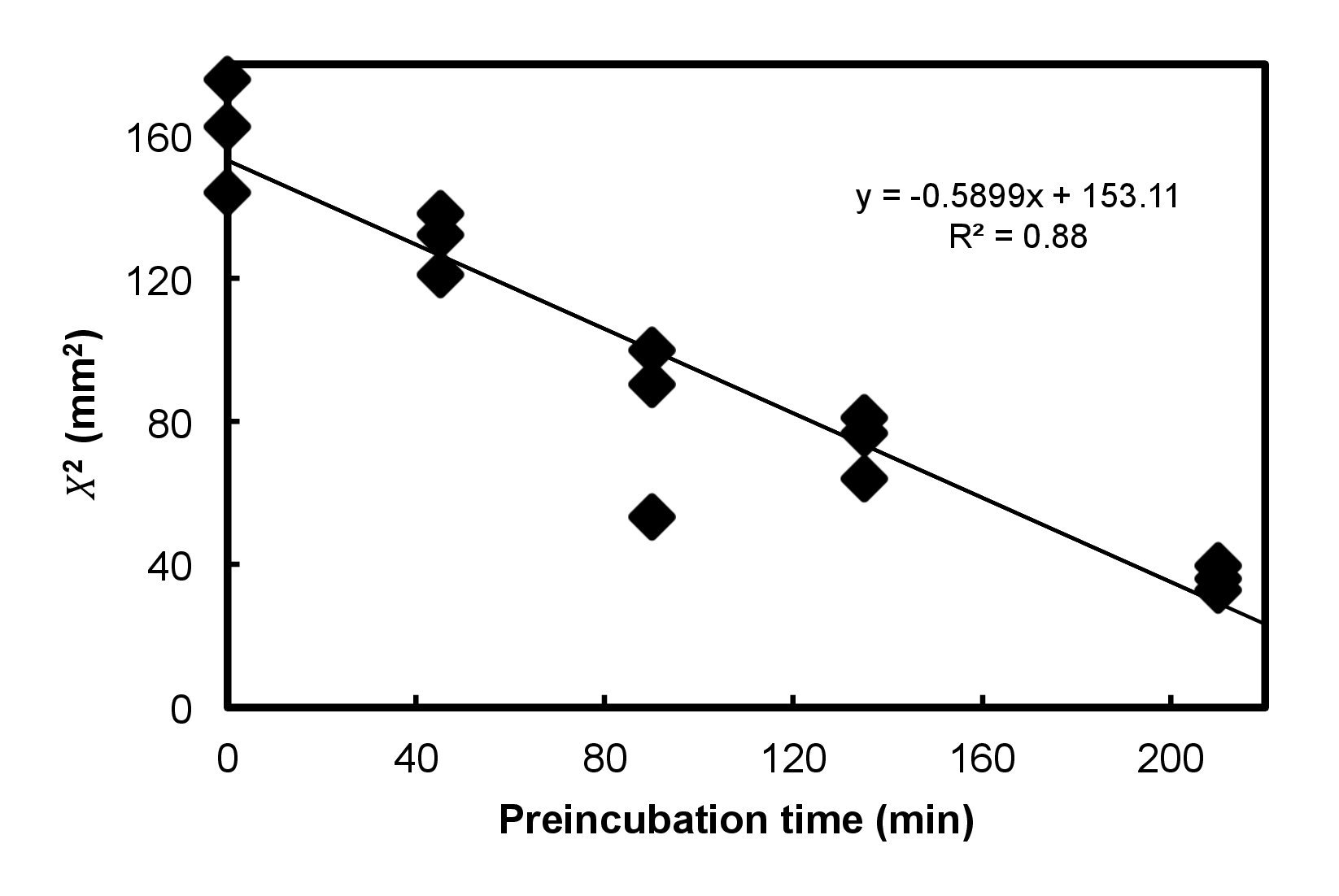

Supplement: S4 Fig — (TIFF) [file pbio.1002044.s004.tiff]
