# Supplementary material for: A Low-Cost, Hands-on Module to Characterize Antimicrobial Compounds Using an Interdisciplinary, Biophysical Approach
Source: PLoS Biol. 2015 Jan 20;13(1):e1002044. doi: 10.1371/journal.pbio.1002044 (PMC4300086; doi:10.1371/journal.pbio.1002044)
Supplement: S1 Table — (DOCX) [file pbio.1002044.s005.docx]

**Table S1**

| **Relative concentration** | $X$ **(mm)** |
| --- | --- |
| 1 | 13 |
| 1 | 16.75 |
| 1 | 14.5 |
| 0.1 | 11.25 |
| 0.1 | 10.5 |
| 0.1 | 13.5 |
| 0.01 | 6 |
| 0.01 | 6 |
| 0.01 | 5.5 |
| 0.001 | 2 |
| 0.001 | 2 |
| 0.001 | 2 |
