# Supplementary material for: A Low-Cost, Hands-on Module to Characterize Antimicrobial Compounds Using an Interdisciplinary, Biophysical Approach
Source: PLoS Biol. 2015 Jan 20;13(1):e1002044. doi: 10.1371/journal.pbio.1002044 (PMC4300086; doi:10.1371/journal.pbio.1002044)
Supplement: S3 Table — (DOCX) [file pbio.1002044.s007.docx]

**Table S3**

| **Relative Concentration** | $X$ **(mm)** |
| --- | --- |
| 1 | 15.5 |
| 1 | 14.5 |
| 1 | 14.3 |
| 0.1 | 11.25 |
| 0.1 | 10 |
| 0.1 | 10 |
| 0.01 | 9.25 |
| 0.01 | 8 |
| 0.01 | 8.5 |
| 0.001 | 4 |
| 0.001 | 4 |
| 0.001 | 4.25 |
| 0.0001 | 0 |
| 0.0001 | 0 |
| 0.0001 | 0 |
| 0.00001 | 0 |
| 0.00001 | 0 |
| 0.00001 | 0 |
