# Supplementary material for: A Low-Cost, Hands-on Module to Characterize Antimicrobial Compounds Using an Interdisciplinary, Biophysical Approach
Source: PLoS Biol. 2015 Jan 20;13(1):e1002044. doi: 10.1371/journal.pbio.1002044 (PMC4300086; doi:10.1371/journal.pbio.1002044)
Supplement: S4 Table — (DOCX) [file pbio.1002044.s008.docx]

**Table S4**

| **Time (min)** | $X$ **(mm)** |
| --- | --- |
| 0 | 12.75 |
| 0 | 13.25 |
| 0 | 12 |
| 45 | 11.5 |
| 45 | 11 |
| 45 | 11.75 |
| 90 | 10 |
| 90 | 7.3 |
| 90 | 9.5 |
| 135 | 8 |
| 135 | 8.75 |
| 135 | 9 |
| 210 | 6.3 |
| 210 | 6 |
| 210 | 5.75 |
