# Supplementary material for: A Low-Cost, Hands-on Module to Characterize Antimicrobial Compounds Using an Interdisciplinary, Biophysical Approach
Source: PLoS Biol. 2015 Jan 20;13(1):e1002044. doi: 10.1371/journal.pbio.1002044 (PMC4300086; doi:10.1371/journal.pbio.1002044)
Supplement: S1 Text — This handout was made available to participants before the hands-on session, using Google Drive. It contains more detailed information than we were able to go into during the 3-hour laboratory session, as well as links to all videos. (DOCX) [file pbio.1002044.s009.docx]

**Characterizing antimicrobial compounds:**

**Using microbiology experiments and an analytical model**

The global emergence and spread of antibiotic resistance is a major clinical and public health problem [1-3]. The increased resistance to current antibiotics and failure to develop new antibiotics has prompted the urgent search for novel antimicrobial agents [4-9].

**Goal of this hands-on session:** To assay the antimicrobial effects of compounds using the disc diffusion technique and apply an analytical model to determine the diffusion coefficient of the active antimicrobial component. The diffusion coefficient depends on size and is therefore a measure of the molecular weight of the active ingredient.

**Section 1: Preparation of equipment and bacterial growth media**

**Preparation of equipment**

1. Equipment to be procured from a commercial source

Pre-sterile petri dishes (alternatively, glass petri dishes may be cleaned and sterilized by autoclaving)

LB broth and agar (or individual components–tryptone, yeast extract and sodium chloride, and agar)

Pre-sterile 50 mL plastic falcon tubes

Pre-sterile plastic pipettes (3 mL, with 0.5 mL graduations) or automated pipettes with tips (1-10 μL, 10-100 μL). For the automated pipettes, tips can be bought pre-sterile or sterilized in the autoclave.

Pre-sterile L-cell spreaders (alternatively, a metal, reusable spreader can be used and sterilized by flaming with 70% ethanol)

Glassware for making and storing LB broth

Analytical balance (to weigh constituents of LB broth and agar)

2. Equipment to be sterilized prior to use

Filter discs (7 mm diameter, Whatman filter paper no. 1) (Video S4)

Wooden sticks (for inoculation of bacterial cultures) (Video S3)

Metal forceps (Video S3)

Pipette tips (if using automated pipettes) (Video S3)

Glass test tubes (if available, for inoculating bacterial broth cultures)

The above equipment was sterilized using an autoclave (121°C for 20 min at 15 psi) with a drying cycle. Alternatively, 70% ethanol and flame sterilization can be used.

**Preparation of bacterial growth media** (Video S1 and S2)

Luria-Bertani (LB) media [10, 11] is routinely used in microbiology laboratories to support bacterial growth. To prepare the liquid or broth form of this media, 10g tryptone, 5g yeast extract, 10g sodium chloride are dissolved in 500 mL distilled water, aliquoted into clean glass bottles and sterilized by autoclaving – *i.e*., by heating to 121°C for 20 min at 15 psi. To make LB agar, 6g of the solidifying agent agar is added to the above constituents before they are autoclaved. This gives an agar concentration of 1.2%. After sterilization by autoclaving, the molten LB agar is poured into pre-sterile petri dishes and allowed to set. Agar plates are then packed into plastic sleeves for storage at 4°C. It is preferable not to use plates more than 3-4 weeks old. Alternatively, instead of individual constituents, commercially available mixed formulations, or ready-to-use forms of LB broth or agar may also be used.

**Section 2: Inoculation and growth of bacterial cultures**

Materials

Pre-sterile 50 mL plastic falcon tubes

Pre-sterile plastic pipettes (3 mL, with 0.5 mL graduations)

Sterile wooden sticks

Sterile LB broth and LB agar plates

Three days before the day of the experiment, the bacterial strain *Escherichia coli* DH5$\alpha$ was streaked onto a plate of LB agar and allowed to grow at room temperature for 48 hours. The bacterial strain was shipped in soft agar (0.6%) in plastic cryovials to the school (Video S5). Bacterial cultures are put up from these plates. The bacterial strain *E. coli* DH5$\alpha$ is considered non-pathogenic to humans. Classified in Risk Group 1 (Biosafety level (BSL) 1) [12, 13] and posing minimum risk to humans, this strain can be worked with on a laboratory bench top using an open flame and requires no additional precautions. It is important to note, that these guidelines apply only to BSL 1 organisms. For working with organisms that are not BSL 1, please refer to the Center for Disease Control (CDC) biosafety guidelines [12, 13].

Procedure (Video S6)

Inoculation of bacterial cultures should be done near a flame using sterile technique. Using a pre-sterile plastic, disposable pipette (or automated pipette, if available), add 10 mL of LB broth into a 50 mL plastic Falcon tube or sterile glass tube. With a wooden stick pick a bacterial colony from the surface of the agar plate and gently suspend the colony into the LB broth. Loosely cap the plastic tube and incubate at room temperature for 24 hours.

**Section 3: Preparation of lawns of bacterial growth**

Materials needed

Overnight bacterial cultures grown in LB broth (from Section 2)

Pre-sterile plastic pipettes (3 mL, with 0.5 mL graduations)

LB agar plates (if stored at 4°C, allow to warm and dry at room temperature prior to use)

Pre-sterile L-cell spreaders

Procedure (Video S7)

Preparation of bacterial lawns should be done near a flame using sterile technique. Using a pre-sterile plastic pipette (3 mL, with 0.5 mL graduations), add 2 large drops (~100 μL) of the bacterial culture grown in LB broth onto the surface of a warm, dry LB agar plate. Using an L-cell spreader gently spread the drop of bacterial culture over the entire surface of the agar. It is advisable to hold the L spreader in the dominant hand and agar plate in the non-dominant hand. Rotating the plate gently, make sure to cover the entire agar surface well including the center and rim. Spread thoroughly, repeating strokes several times. After spreading is complete, allow the plates to dry for a few minutes.

**Section 4: Deposition of antimicrobial compounds**

Materials needed

Bacterial lawns spread on the surface of LB agar (from Section 3)

Sterile filter discs (7-mm diameter)

Sterile metal forceps

Antimicrobial test compounds

We will be testing three compounds – the natural extract eucalyptus oil (100%, *Eucalyptus globulus*) [14-19], and the chemical compounds ethanol (70%) and hydrogen peroxide (3%) [20]. Eucalyptus oil was procured from NOW® Solutions and ethanol and hydrogen peroxide were obtained from a local pharmacy in Trieste, Italy.

Procedure (Video S7)

Using a metal forceps place a filter disc (7-mm diameter) on the surface of the bacterial lawn. Using a plastic, disposable pipette, add a drop (~50 μL) of eucalyptus oil or ethanol or hydrogen peroxide on the filter disc, minimizing the amount of spillover around the disc as much as possible. As a control, deposit a sterile filter disc onto the lawn but do not apply any antimicrobial compound. Allow the antimicrobial compounds to dry and incubate plates at room temperature for 24-48 hours. Make sure to label each plate appropriately using a Sharpie pen. This technique is called the ‘disc diffusion’ assay [21, 22] and is routinely used in clinical and research microbiology laboratories to study the antibiotic susceptibility of bacterial strains [23, 24].

**Section 5: Evaluating the efficacies of the antimicrobial compounds**

Materials needed

Experiment plates from Section 4

Ruler

Procedure

Following incubation at room temperature for 24-48 hours, antimicrobial efficacy is observed as ‘zones of inhibition’ of the bacterial lawn around the filter discs (Figure 2). Using a ruler, measure the size of the zones of inhibition from the edge of the filter disc to the edge of the zone. We refer to this width of the inhibition zone as $X$. Alternatively, plates can be photographed and the zones of inhibition can be measures using an open-source, image analysis software (such as ImageJ) [25]. By measuring the size of the zone of inhibition, the efficacies of different antimicrobial compounds and the susceptibility of different bacterial strains can also be compared.

**Section 6: Using an analytical model to determine the physical characteristics of the active ingredient of an antimicrobial compound**

To determine the physical properties of the active ingredient of a compound ($I$) exhibiting antimicrobial activity, our laboratory has developed a numerical model based on the disc-diffusion assay [26, 27].

The assumptions of the model include –

1. The active ingredient of the antimicrobial compound is released at a concentration $C_{0}$ at the filter disc.
2. The active ingredient diffuses out of the disc with a constant diffusion coefficient $D$.
3. A threshold concentration of the active ingredient is required to inhibit bacterial cells.
4. The active ingredient no longer effects inhibition after a critical time $T_{c}$ of incubation.

Assumption (4) corresponds an increase in the number of cells in the lawn that causes the per-cell concentration of the active ingredient to drop to sub-inhibitory levels.

Based on this model,

$X^{2}=4DT_{c}\ln(C_{0})+F(D,T_{c},C_{c})$ (1)

where $C_{0}$ is the concentration of the antimicrobial compound deposited on the filter disc, which we assume to be linearly proportional to the concentration of the active ingredient. $F$ is a function independent of $C_{0}$. $C_{c}$ corresponds to the lowest concentration of the active ingredient required to cause measurable inhibition. $T_{c}$ is the critical time of incubation (also called pre-incubation time) after which the inhibition is no longer observed. At this time, the density of the bacterial lawn increases to a critical level (due to bacterial growth), resulting in the concentration of the active ingredient falling below the critical threshold concentration required to effect inhibition. As seen in equation 1, the slope of $X^{2}$ (square of the width of inhibition) as a function of $\ln(C_{0})$gives the diffusion coefficient $D$, if critical time $T_{c}$ is known.

Further, using the calculated diffusion coefficient $D$, we can determine the molecular weight $(MW)$. According to the Stokes-Einstein equation [28], the diffusion coefficient of a molecule is inversely proportional to its radius.

From the equations,

$D=\frac{\kappa_{B}T}{6\pi\eta R}$ (2)

where is $\kappa$ Boltzman constant, $T$is temperature, $\eta$ is solvent viscosity and $R$ is radius of the molecule,

and $MW=N\rho V=N\rho*\frac{4}{3}\pi R^{3}$ (3)

where $N$ is Avogadro’s number, $\rho$ is density of the molecule, $V$ is volume of the molecule, and $R$ is radius of a spherical molecule, we get that $D\propto\frac{1}{MW^{1/3}}$

To first order, a molecule’s volume and therefore its molecular weight is proportional to its radius, thus for the active ingredient of antimicrobial compound $I$ and a known molecule $A$:

$MW_{I}=MW_{A}(\frac{D_{A}}{D_{I}})^{3}$ (4)

Owing to insufficient time, experiments to measure the slope of $X^{2}$ (square of the width of inhibition) as a function of $\ln(C_{0})$and pre-incubation time $T_{c}$ are demonstrated in these video clips (Video S8 and S9). To measure the slope of $X^{2}$ as a function of $\ln(C_{0})$ decreasing concentrations of the antimicrobial compound are deposited on bacterial lawns and after overnight incubation, the width of inhibition ($X$) for each concentration is measured. Using linear regression, the slope of $X^{2}$ as a function of $\ln(C_{0})$ is obtained. To measure pre-incubation time, a given concentration of the antimicrobial compound is deposited on the bacterial lawn after different time intervals of incubation. After overnight incubation, the width of inhibition ($X$) for each time point is measured. Using linear regression, the time after which no inhibition is observed ($X$ = 0 mm) is determined as the critical time of pre-incubation.

**Section 7: Analysis of data to determine diffusion coefficient** $D$ **and molecular weight (**$MW$**) of the active ingredient of compound** $I$

To provide representative data sets for analysis, we performed the above experiments with hydrogen peroxide (compound $I$) and a known antibiotic tobramycin ($MW$= 467.5 Da).

**Raw Data for compound $I$(hydrogen peroxide)**

Increasing concentrations of compound $I$ (hydrogen peroxide) and corresponding sizes of zones of inhibition ($X$) (Table S1).

**Hint:** Use this to obtain the slope of $X^{2}$as a function of $\ln(C_{0})$ as seen in equation (1).

Increasing time of pre-incubation and corresponding sizes of the zones of inhibition ($X$) for compound $I$(hydrogen peroxide) (Table S2).

**Hint:** Use this data to obtain the critical pre-incubation time $T_{c}$ of the active ingredient of compound $I$as seen in equation (1).

Using the slope and value of $T_{c}$ obtained above, calculate the diffusion coefficient $D$ of the active ingredient of compound $I$.

**Raw Data for known compound** $A$

The known antibiotic tobramycin ($MW$= 467.5 Da) was used as the standard to calculate the molecular weight of the active ingredient of compound $I$.

Increasing concentrations of tobramycin and corresponding sizes of zones of inhibition ($X$) (Table S3).

Increasing time of pre-incubation ($T_{c}$) and corresponding sizes of the zones of inhibition ($X$) for tobramycin (Table S4).

Using the slope and value of $T_{c}$ obtained above, calculate the diffusion coefficient $D$ of tobramycin.

Using the value of the diffusion coefficients for compound $I$ and tobramycin calculated above and the known molecular weight of tobramycin ($MW$ 467.5 Da), obtain the molecular weight of the active ingredient of compound $I$ (refer equation 3).

**Important to note:** The values of $D$ and $T_{c}$ obtained above depend on the bacterial strain, media and incubation conditions. It is therefore essential that the experiments for the test antimicrobial compound and known molecular weight standard be done under identical conditions.

**Section 8: Solution to data analysis in Section 7.**

**For compound** $I$

Slope of $X^{2}$as a function of $\ln(C_{0})$ for compound $I$(Figure S1).

Using linear regression, we get the slope of $X^{2}$ as a function of $\ln(C_{0})$ (y = ax+ b).

Pre-incubation time $T_{c}$ for compound $I$ (Figure S2).

Using linear regression, the pre-incubation time (y-intercept) is determined as 301 ± 39 minutes.

From the above values we get the diffusion coefficient $D$ of the compound$I$as 4.5 ± 0.8 X 10^-6^ cm^2^/sec.

**For Tobramycin**

Slope of $X^{2}$ as a function of $\ln(C_{0})$ for tobramycin (Figure S3).

Using linear regression, we get the slope of$X^{2}$ as a function of $\ln(C_{0})$ (y = ax+ b).

Pre-incubation time $T_{c}$ for tobramycin (Figure S4).

Using linear regression, the pre-incubation time (y-intercept) is determined as 260 ± 28 minutes.

From the above values we get the diffusion coefficient $D$ of tobramycinas 2.9 ± 0.5 X 10^-6^ cm^2^/sec.

Using $MW_{I}=MW_{A}(\frac{D_{A}}{D_{I}})^{3}$ and the known molecular weight of tobramycin as 467.5 Da, we get the molecular weight of the active ingredient of compound $I$as 128 ± 96 Da. This is consistent with the molecular weight of hydrogen peroxide which is 34 Da. Based on our module, we can conclude that the antimicrobial component is a small, low-molecular weight compound, approximately 32-224 Da in size.

**References**

1. Levy SB (2002). Factors impacting the problem of antibiotic resistance. J Antimicrob Chemother 49: 25-30.
2. Höjgård S (2012) Antibiotic resistance – why is the problem so difficult to solve? Infect Ecol Epidemiol 2: 10.3402/iee.v2i0.18165.
3. Davies J, Davies D (2010) Origins and evolution of antibiotic resistance. Microbiol Mol Biol Rev 74: 417-433.
4. Friere-Moran L, Aronsson B, Manz C, Gyssens IC, So AD, et al. (2011) Critical shortage of new antibiotics in development against multidrug-resistant bacteria – Time to react is now. Drug Resistant Updates 14: 118-124.
5. Jagusztyn-Krynicka EK, [Wyszyńska A](http://www.ncbi.nlm.nih.gov/pubmed?term=Wyszy%C5%84ska%20A%5BAuthor%5D&cauthor=true&cauthor_uid=18646395) (2008) The decline of antibiotic era – new approaches for antibacterial drug discovery. Pol J Microbiol 57: 91-98.
6. Luzhetskyy A, Pelzer S, Bechthold A (2007) The future of natural products as a source of new antibiotics. Curr Opin Invest Drugs 8: 608-613.
7. Villa F, Villa S, Gelain A, Cappitelli F (2013) Sub-lethal activity of small molecules from natural sources and their synthetic derivatives against biofilm forming nosocomial pathogens. Curr Top Med Chem 13: 3184-3204.
8. Brown DG, Lister T, May-Dracka TL (2014) New natural products as new leads for antibacterial drug discovery. Bioorg Med Chem Lett 24: 413-418.
9. Prabuseenivasan S, Jayakumar M, Ignacimuthu S (2006) In vitro antibacterial activity of some plant essential oils. BMC Complem Altern M 6: 39.
10. Bertani G (1951) Studies on lysogenesis. I. The mode of phage liberation by lysogenic Escherichia coli. J Bacteriol 62: 293-300.
11. Experiments in molecular genetics (1972) In: Miller JH, editor. New York: Cold Spring Harbor Laboratory, Cold Spring Harbor Lab. Press.
12. Center for Disease Control and Prevention, Biosafety. Available at: http://www.cdc.gov/biosafety/publications/bmbl5/bmbl5_sect_iv.pdf (Accessed 2 September 2014).
13. Center for Disease Control and Prevention, Biological Risk Assessment. Available at:

http://www.cdc.gov/biosafety/publications/bmbl5/bmbl5_sect_ii.pdf (Accessed 2 September 2014).

1. Bachir RG, Benali M (2012) Antibacterial activity of the essential oils from the leaves of Eucalyptus globulus against Escherichia coli and Staphylococcus aureus. Asian Pac J Trop Biomed 2: 739-742.
2. Karpanen TJ, Worthington T, Hendry ER, Conway BR, Lambert PA (2008) Antimicrobial efficacy of chlorhexidine digluconate alone and in combination with eucalyptus oil, tea tree oil and thymol against planktonic and biofilm cultures of Staphylococcus epidermidis. J Antimicrob Chemother 62:1031-1036.
3. Hendry ER, Worthington T, Conway BR, Lambert PA (2009) Antimicrobial efficacy of eucalyptus oil and 1,8 – cineole alone and in combination with chlorhexidine digluconate against microorganisms grown in planktonic and biofilm cultures. J Antimicrob Chemother 64: 1219-1225.
4. Trivedi NA, Hotchandani SC (2004) A study of the antimicrobial activity of oil of Eucalyptus. Indian J Pharmacol 36: 93-95.
5. Inouye S, Takizawa T, Yamaguchi H (2001) Antibacterial activity of essential oils and their major constituents against respiratory tract pathogens by gaseous contact. J Antimicrob Chemother 47: 565-573.
6. Elaissi A, Rouis Z, Ben Salem NA, Mabrouk S, ben Salem Y, et al (2012O Chemical composition of 8 eucalyptus species’ essential oils and the evaluation of their antibacterial, antifungal and antiviral activities. BMC Complem Altern M 12: 81.
7. McDonnell G, Russell AD (1999) Antiseptics and disinfectants: activity, action and resistance. Clin Microbiol Rev 12: 147-179.
8. Bauer AW, Kirby WMM, Sherris JC, Turck M (1966) Antibiotic susceptibility testing by a standardized single disk method. Am J Clin Pathol 45: 493-496.
9. Kirby WMM, Yoshihara GM, Sundsted KS, Warren JH (1957) Clinical usefulness of a single disc method for antibiotic sensitivity testing. Antibiotics Annu 1956-1957:892-897.
10. Jorgensen JH, Turnidge JD (2007) Antibacterial susceptibility tests: dilution and disk diffusion methods. In: Murray PR, Baron EJ, Jorgensen JH, Landry ML, Pfaller MA, editors. Manual of Clinical Microbiology. Washington, DC: American Society for Microbiology. pp. 1152-1172.
11. Jorgensen JH, Ferraro MJ (2009) Antimicrobial susceptibility testing: A review of general principles and contemporary practices. Clin Infect Dis 49: 1749-1755.
12. Abramoff MD, Magalhaes PJ, Ram SJ (2004) Image Processing with ImageJ. Biophotonics International 11: 36-42.
13. Cooper KE (1955) Theory of antibiotic inhibition zones in agar media. Nature 176: 510-511.
14. Cooper KE (1963) The theory of antibiotic inhibition zones. In: Kavanagh F editor. Analytical Microbiology. New York: Academic press. pp. 13-30.
15. Edward JT (1970) Molecular volumes and the Stokes-Einstein equation. J Chem Educ 47: 261-270.
